# The Patient’s Perspective of in-Home Telerehabilitation Physiotherapy Services Following Total Knee Arthroplasty

**DOI:** 10.3390/ijerph10093998

**Published:** 2013-08-30

**Authors:** Dahlia Kairy, Michel Tousignant, Nancy Leclerc, Anne-Marie Côté, Mélanie Levasseur, the Telage Researchers

**Affiliations:** 1 School of Rehabilitation, Université de Montréal and Centre for Interdisciplinary Research in Rehabilitation of Greater Montreal—IRGLM site, 6300 Darlington Avenue, Montreal, Quebec H3S 2J4, Canada; 2 Research Centre on Aging, University Institute of Geriatrics of Sherbrooke, Faculty of Medicine and Health Sciences, Université de Sherbrooke, 1036 Belvédère Sud, Sherbrooke, Quebec J1H 4C4, Canada; E-Mails: michel.tousignant@usherbrooke.ca (M.T.); nancy.leclerc@usherbrooke.ca (N.L.); 3 School of Rehabilitation, Faculty of Medicine and Health Sciences, Faculty of Medicine and Health Sciences, Université de Sherbrooke, 1036 Belvédère Sud, Sherbrooke, Quebec J1H 4C4, Canada; E-Mails: anne-marie.cote3@usherbrooke.ca (A.-M.C.); Melanie.levasseur@usherbrooke.ca (M.L.)

**Keywords:** satisfaction, telerehabilitation, patients, semi-structured interview, qualitative

## Abstract

This study aimed at exploring patients’ perceptions regarding telerehabilitation services received post total knee replacement. In this qualitative embedded single case study, semi-structured interviews were conducted with five patients who had previously received in-home telerehabilitation post total knee arthroplasty. Participants were asked to reflect on their 8-week rehabilitation process and on their experience with the home telerehabilitation program. Interviews were transcribed and a qualitative thematic analysis was conducted. Six overarching themes emerged from the patients’ perceptions: (1) improving access to services with reduced need for transportation; (2) developing a strong therapeutic relationship with therapist while maintaining a sense of personal space; (3) complementing telerehabilitation with in-person visits; (4) providing standardized yet tailored and challenging exercise programs using telerehabilitation; (5) perceived ease-of-use of telerehabilitation equipment; and (6) feeling an ongoing sense of support. Gaining a better understating of the patient’s experience in telerehabilitation will be essential as programs continue to be developed and implemented.

## 1. Introduction

Important demographic changes, including an aging population, increased life expectancy and a greater prevalence of chronic conditions are putting increased strain on health care systems worldwide. Moreover, the reduced length of hospital stays implies that patients return home sicker and with incapacities [1,2,3]. To better meet these changing needs, different modes of health service delivery have been proposed and developed. For example, home care services are well implemented in Canada. However, it is recognized that home care cannot respond to the increasing demand for services [4,5] and the lack of human resources [6,7]. Consequently, in-home telehealth, including telerehabilitation programs, are becoming increasingly common as an alternative mode of service delivery. Telerehabilitation is defined as the provision of rehabilitation services at a distance using information and communication technologies [8,9,10].

Several studies have examined in-home telerehabilitation programs. Initial studies confirmed the technical feasibility of in-home telerehabilitation [11,12,13]. More recent studies explored the efficacy of such services in many patient populations and a number of systematic reviews and reports have summarized these findings [8,14,15,16,17].

The successful implementation and integration of telehealth programs, including home telehealth, remains slow [18]. It is increasingly recognized that the patient’s perception of the services should be taken into account when implementing telehealth, including home telerehabilitation [19]. Despite recognizing the importance of the patient’s perspective, it remains absent from many studies [20], as was similarly reported by Mair *et al.* in 2000 [21]. In telehealth, the patient’s perspective is generally documented through the concept of satisfaction and is reported using questionnaires and surveys, primarily addressing the technical aspects of using the technology and the communication between the participants [22].

In telerehabilitation, a similar trend has been noted [17]. For example, a previous study conducted reported on satisfaction with in-home telerehabilitation for patients and health professionals following a randomized clinical trial (RCT) post Total Knee Arthroplasty (TKA) [23]. High satisfaction rates were noted with three predetermined factors: the technology, the health care services and the relationship with the health care professional using validated questionnaires. The results obtained from this quantitative study [23] were limited to the participants’ satisfaction concerning exclusively these three factors (percentage).

Few studies have reported on aspects other than satisfaction when considering the patient’s point-of-view. Two studies examined chronic pain sufferers’ perspective of in-home telerehabilitation. The first used semi-structured interviews with participants who were informed about possible telerehabilitation services [24]. The second used a questionnaire based on the Technology Acceptance Model (TAM) with participants randomly allocated either to an experimental group who got information about and could try out a web-based program which provided selected instructional videos or a control group who only got information about the web-based program [25]. In the first study [24], the authors reported that patients appreciated the flexibility that telerehabilitation could provide, but were concerned with the lack of therapist in-person contact on their ability to successfully participate in an exercise program. In the subsequent study [25], participants were more positive about the usefulness and ease-of-use of the telemedicine program after experiencing it than before. Thus, brief use of telemedicine has a significant positive effect on participants’ perception of the technology.

Similarly, in a chronic pain population [26], participant satisfaction (perceived usefulness, ease of use and intent to use) was documented using a questionnaire based on the TAM, following a myofeedback-based teletreatment which recorded data that was transmitted to the therapist for a weekly teleconsultation. The authors reported that for a majority of participants, perceived ease of use and usefulness increased after using the technology, although this study did not show a relationship between satisfaction, compliance with treatment and clinical outcome.

Eriksson *et al*. [27] reported on the experience of patients in Sweden who had recently experienced in-home telerehabilitation following a shoulder joint replacement using interviews with the patients. Participants reported “feeling close at a distance” with their therapist who was able to guide them in a home exercise program, overcoming their fear of pain.

Hence, few studies have explored the patient’s perspective of regarding telerehabilitation, even though patients’ perceptions can have a significant impact on rehabilitation outcome [28]. This study aimed at better understanding the patient’s experience of home telerehabilitation. More specifically, this study explored the perception of patients who have undergone a total knee replacement (TKA) concerning in-home telerehabilitation services.

## 2. Methods

### 2.1. Study Design

An embedded single case study design [29] was used in order to obtain an in-depth understanding of the patients’ perception of the actual telerehabilitation services received. This type of study design facilitates the understanding of the phenomenon of in-home telerehabilitation post TKA that is at the same time context-dependent and influenced by the individual patient’s experience and characteristics [29,30]. The case analysed was an in-home telerehabilitation program for patients who had undergone total knee arthroplasty (see Section 2.3).

### 2.2. Participant Recruitment

In order to recruit participants who had experienced telerehabilitation and given the lack of actual home telerehabilitation programs, patients were selected from a pool of participants from the experimental arm of a RCT for in-home telerehabilitation post-TKA. In this context, we used non-probability sampling. Participants were recruited once they had fully completed their participation in the study, so as not to impact on the RCT. This study was not designed as part of the RCT TKA (RCT recently concluded and findings not yet published). In order to obtain an in-depth understanding of the phenomena, a purposive sample was selected, as suggested by Groenewald [31]. In order for participants to be able to comment on their telerehabilitation experience, and compare it to in-person types of physiotherapy services, only participants who previously received physiotherapy services in the community, including but not limited to a previous TKA, were recruited. Eligible patients were invited to take part in an in-person interview concerning their experience with the in-home telerehabilitation service. This study was approved separately from the RCT by the appropriate ethics review boards, with informed consent obtained from all participants.

### 2.3. Description of the in-Home Telerehabilitation Program

**Figure 1:**
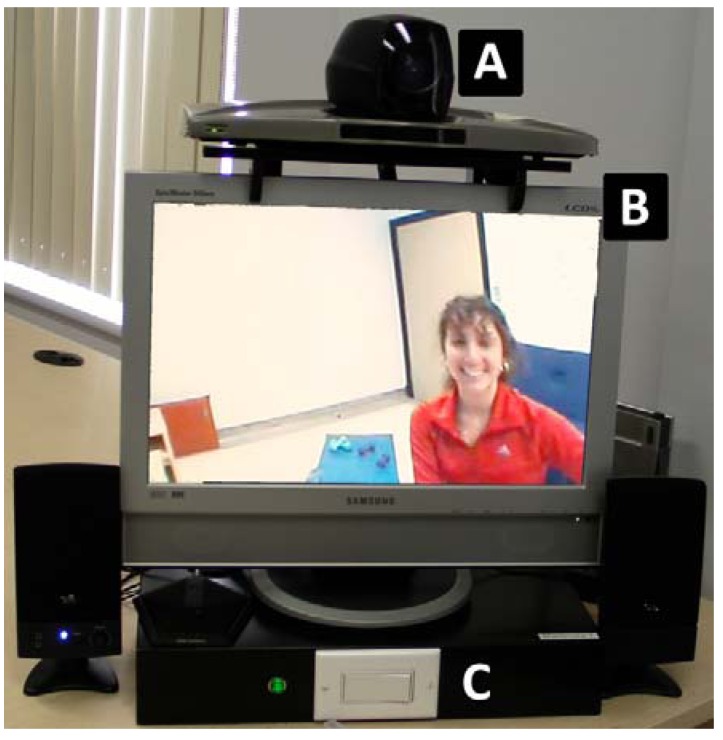
Set-up for in-home telerehabilitation: (A) Videoconferencing system; (B) Screen; (C) on/off switch.

The in-home telerehabilitation program consisted of twice-a-week physiotherapy sessions for eight weeks (total 16 sessions), each session lasting 45 to 60 min. The content of the intervention was an adaptation of the Intensive Functional Rehabilitation (IFR) protocol [32]. Clinical equipment (step, exercise pedal, 3- and 5-pounds weights and elastics) was lent out to each participant for the entire duration of the intervention. The intervention was aimed at improving walking and functional autonomy in daily activities as well as mobility and strength of lower limbs. A videoconferencing system located in the participant’s home was connected remotely through high speed internet to the health center’s system where the physiotherapist was located. The telerehabilitation platform used was developed with a user-friendly system to ensure that interaction between clinician and patient during the session was similar to that of the in-person intervention (Figure 1). The platform is the same as the one used for previous studies [11,33]. Prior to starting the telerehabilitation services, participants had never met their therapist in person.

### 2.4. Data Collection and Analysis

A semi-structured individual interview was conducted with each patient in their home by a research agent who is a member of our research team and had not been involved in providing the patients’ telerehabilitation services. An interview guide was developed based on the literature in the area of technology adoption which suggests that concepts such as perceived usefulness, perceived ease of use, attitude, intent to use, actual use and other external variables will affect how technology is used [34]. Participants were asked to reflect on their entire rehabilitation experience, with a particular emphasis on key events such as finding out they would receive services at home by videoconference, having the internet and videoconferencing equipment installed at home and receiving services by videoconference including dealing with technical issues and completing the 8-week rehabilitation process.

Interviews were audiorecorded and transcribed word for word. Each transcript was read and coded line by line by one member of the research team using Text Analysis Markup System (TAMS) Analyzer. We used investigators triangulation such that the interviews were initially analysed by one researcher and then reread and confirmed by another researcher. When they had differences in the codification, the researchers arrived at a consensus. The findings were then discussed in group with the research team thus increasing the validity of the findings. Similarities and differences between the cases were noted throughout the coding process and overarching themes were identified by the research team [35].

In order to analyse each unit of analysis or each patient’s perception, we used thematic analysis by Miles and Huberman [35] as mentioned by Yin [29]. This method provides an exhaustive description of each unit of analysis (own patient perception of the telerehabilitation). We then used matrices to explain the whole case and compare between patients to obtain a broader perception of in-home telerehabilitation.

## 3. Results

Participant characteristics that could impact on the patient’s experience of telerehabilitation (e.g., gender, age, work status, type of housing, living alone or not, internet access that was previously installed or not, functional status prior to surgery) are presented in Table 1. Interviews lasted on average 55 min, ranging from 37 to 67 min.

Overall, all participants agreed that the telerehabilitation treatment was a good alternative to in-person physiotherapy sessions. Upon analysis, the patients’ perceptions of in-home telerehabilitation services post-TKA were regrouped into six main themes as reported in the following section. Verbatim quotes illustrate the results. Following the analysis in the original French Canadian version, quotes were translated into English for publication, while retaining the style and meaning of the quote.

### 3.1. Improving Access to Services

The predominant benefit mentioned by all participants, was the elimination of all transportation time for both the patient and therapist. More specifically, this was viewed as particularly useful the first few weeks post-surgery when participants had more pain, as compared to travelling for out-patient services.

**Table 1:** Patients’characteristics.

Characteristics	Participant 1	Participant 2	Participant 3	Participant 4	Participant 5
Gender	Woman	Man	Man	Woman	Woman
Age (years)	44	72	62	59	70
Years of schooling (years)	15	18.5	15	16	11
Work status	Not working	Retired	On disability	Specialized educator (on leave for recovery from TKA)	Retired
Living alone	Yes	No, with wife	Yes	Yes	No, with husband
Type of housing	Apartment building	Single family dwelling	Single family dwelling	Apartment building	Duplex
Stairs at home	Yes	Yes	No	Yes	Yes
Prior internet service	No	Yes	No	Yes	No
Years of knee pain (years)	1–5	More than 10	1–5	More than 10	More than 10
Other medical condition	Arthritis, diabetes, anxiety, disc degeneration	Arthritis, diabetes, gastro-intestinal disorder, visual problem, hypertension, prostate cancer in remission	Arthritis, asthma, acute respiratory distress syndrome (ARDS), angina, gastro-intestinal disorder, depression, anxiety	Arthritis, asthma, diabetes, depression, anxiety, sleep apnea, hypertension	Arthritis, gastro-intestinal disorder, hyperthyroidism, double pulmonary embolism

“I really like it (telerehabilitation). I found it fantastic…you know, just the fact of not having to travel when we are in pain (…) I adored it…” (participant 2)

Decreased preparation time was reported as a benefit, in particular not having to get ready to attend an appointment in an out-patient setting. However, two of the participants did not feel that they needed to save time at that particular point in their rehabilitation, when they are not working or participating in many activities. Nevertheless, they do consider that telerehabilitation would be of benefit to others as, according to them, it allows patients to access health services more easily and therapists to see more patients.

### 3.2. Developing a Bond with Their Therapist While Maintaining a Sense of Personal Space

Although services were not provided face-to-face, all patients appreciated the contact they have with their physiotherapist and their availability. All participants felt they developed a relationship with the physiotherapist who made them at ease to express their needs. Overall, they felt listened to and felt that they could express their concerns regarding their condition or other more personal issues. They perceived the physiotherapist as supportive and well informed about their physical condition. For example, participants reported that they felt as if the therapist was there in person. Moreover, participants appreciated having informal conversations with their therapist. Four of the participants mentioned that the therapist became like a family member.

“Well look, she (the physiotherapist), was roughly my nieces’ age. So it was the same as if I was an aunt with her” (participant 1)

“…we talked about fishing, we talked about hunting, (…) we talked about skiing, hum, of all sorts of things, while I was doing my exercises, we talked about anything and we always had something to say. I think that she knew my whole life (laughter) (…)” (participant 4)

One of these participants even preferred the use of telerehabilitation as compared to having the therapist come to her home in person, such as was the case for participants who were assigned to the usual-care arm of the RCT:

“I was satisfied. (...) the fact that she (the physiotherapist) was not with me in the house, I was less stressed.” (participant 4)

As compared to out-patient services, participants reported that they appreciated the increased sense of privacy and the bond they developed with their therapist with the use of telerehabilitation, as compared to being in a physiotherapy department among other patients and therapists. Four of the participants also felt the use of telerehabilitation provided their therapist with insight into their home environment.

### 3.3. Complementing Telerehabilitation with in-Person Visits

A benefit mentioned by all the participants was the perception that the physiotherapist was able to adequately evaluate via telerehabilitation the amplitude of the knee as well as the scar and their fatigue and pain. They found the intensity of the exercises and length of the sessions to be appropriate for their condition. Despite participants reporting that the use of telerehabilitation was appropriate for their condition, three of the participants felt that their rehabilitation should have included some in-person visits with their therapist. They felt that complementing telerehabilitation with the occasional in-person visit would improve the physiotherapist’s evaluation of the knee as well as facilitate clinical follow-up. For example, two participants mentioned that they would have liked more physical contact with a therapist in order to ensure that they were progressing adequately and to deal with issues that arose, such as poor patellar mobility, in a timelier manner.

“… she would have seen if she had touched me that my patella was not in the right place.” (participant 1)

“I’m fairly certain that at least twice, on two occasions certainly if he would have come, it would have been a plus. Well, maybe psychologically, I think, thinking that he could have manipulated your knee, to see in a tangible manner and be able to manipulate it, but hum… it’s the suggestion that I would give, to at least meet, I don’t know how often … (…).” (participant 2)

In addition, two participants compared the assessment findings recorded by their therapist to those reported by their orthopedist or another therapist. The participant who felt it was important to have in-person visits with the therapist (participant 1) reported that an orthopedist’s assessment of the range of motion differed from that of their therapist. On the other hand, the participant who did not feel it was necessary to have an in-person visit with the therapist reported that their therapist’s findings were identical to those of another therapist.

### 3.4. Providing Standardized yet Tailored and Challenging Exercise Programs Using Telerehabilitation

Although participants followed a standardized exercise program, they all felt their therapist tailored the exercises appropriately thus respecting their fatigue, pain and abilities. They were confident that the therapists provided appropriate supervision from a distance.

Participants appreciated being able to perform some of their exercise program outside of the scheduled therapy time, thus increasing their exercise time with their therapist during the sessions.

“I installed the things I needed. Like that, all my bicycle, and hum... my step. I installed that and it went well. Look, it took 2 min.” (participant 3)

“I got on the bike (stationary bicycle). I was hooked up (by videoconference), and I got on the bike. Instead of him (the physiotherapist) watching me for 10–15 min, I had already done a few minutes. So that after that, well, we did the rest.” (participant 4)

### 3.5. Perceived Ease-of-Use of Telerehabilitation Equipment

The use of new technology was not viewed as a limitation by any of the participants and did not hinder their appreciation for telerehabilitation. All found it easy to use (“the touch of an on/off button”) and found the equipment of little inconvenience in the space it occupied or the change it produced to their home environment. The human aspect surrounding the new technology were positively viewed including the process of setting up the internet connection, installing the technology in the home and the trouble-shooting provided by the telerehabilitation team. Participants reported that they did not feel additional stress when receiving services by telerehabilitation even for the few times when there were communication difficulties. Four participants appreciated the clarity of the sound and the concordance of the voice and the image, although in two of the five cases there were sessions with a delay. All participants reported that the image of the physiotherapist was clear. Despite the freezing of the image reported by one patient, the transmission of the image did not impact on the perception of the quality of the treatment received for that person.

### 3.6. Achieving an Ongoing Sense of Support

At the time of discharge from the hospital, none of the participants were worried about receiving telerehabilitation treatments. They were confident in the telerehabilitation team and considered that the information provided throughout was clear regarding upcoming steps and appointments.

“They had told me that it would be this way (…). So being advised, you know, you’re ok. (…) This way, being advised of the date, that the beginning of the treatments will be on such and such a date. And having the little handouts that said which exercises to do, well then ultimately, it was positive regardless. We say well we’re heading in, in the right direction… to recuperate.” (participant 2)

Participants considered the telerehabilitation technical support team as part of team providing therapy and they all expressed that they felt well supported by the entire team at all times.

## 4. Discussion

In this study, participants were interviewed regarding their telerehabilitation experience post-surgery for a TKA as compared to their previous experience with rehabilitation services. Contrary to the previous quantitative study conducted by our research team about satisfaction of patients concerning telerehabilitation [23], the analyzed themes of the present qualitative research were identified after the interviews and covered a larger spectrum of the patient’s experience. Although participants never met their therapist face-to-face, they felt that their therapist was able to provide a tailored exercise program, adjusting it to according to their ability, pain and fatigue. Participants also felt that they developed a bond with their therapist and felt supported throughout their rehabilitation. None of the participants regarded the videoconferencing technology or the space that it occupied as a barrier to receiving quality rehabilitation services. Three participants did however express a preference for combining telerehabilitation with more traditional in-person services.

All the participants in this study were confident that they participated in an exercise program that was tailored to their needs. These findings differed from that of Cranen *et al*. [24], where patients reported their perception of telerehabilitation after receiving an introduction to in-home telerehabilitation through examples of potential exercise-based telerehabilitation services, without actually experiencing it. They found that participants were concerned with the quality of the feedback they would obtain from their therapist when interviewed regarding *potential* in-home telerehabilitation services for chronic pain. In a subsequent study [25], patients who were randomly allocated to trying out a web-based exercise program for a few minutes were more positive about ease-of-use and usefulness as compared to those who did not actually experience it. Hence, actually experiencing the technology may be important when assessing the patient’s perception of the service.

Eriksson *et al*. [27] also reported on participants’ perception of telerehabilitation, after experiencing it following a shoulder joint replacement. In their study, participants felt they received appropriate feedback and support through *actual* telerehabilitation following a shoulder joint replacement. Thus, the actual experience of telerehabilitation may impact on participants’ perceptions of the ability of a therapist to provide adequate guidance and appropriate exercises from a distance.

An important component of the rehabilitation process is the relationship and trust that develops between the patient and therapist. Crepeau and Garren [36] describe that the therapeutic relationship that emerges between a therapist and a patient who meet in-person develops through the use of humor to establish reciprocity, ordinary conversation to build the rapport and providing attention which is viewed as caring. In our study, all the patients reported discussing elements of ordinary life with their therapist, feeling that their therapist was attentive to their needs throughout, and that a close relationship did in fact develop with their therapist, similar to the findings reported by Eriksson *et al*. [27]. In contrast, in Cranen *et al*.’s study of *potential* telerehabilitation services, patients felt that an emotional bond would not develop with their therapist, and that this could have a negative impact on their rehabilitation outcome [24]. In fact, they expressed that telerehabilitation would make them feel alienated and would be impersonal. Hence, actually experiencing telerehabilitation seems to therefore alter the perception of the patient-therapist relationship that can develop [36].

Some participants in our study felt that telerehabilitation should be complemented by hands-on therapy. Participants expressed that it would be beneficial in order to improve the physical assessment of their knee, namely palpation and range of motion, and in particular when there is contradictory information from different health professionals. The desire for some in-person contact with the therapist was also reported in Cranen’s study [24]. However, in that study, participants considered in-person contact to be important to ensure emotional support, whereas in our study it was viewed as important to improve the physical assessment. The differences between these studies may be the result of changes in perception when participants actually experience telerehabilitation, as mentioned previously. It may also depend on the medical condition or on what patients are comparing the telerehabilitation services to, as rehabilitation services can be provided in a variety of modes, including in and out-patient settings and in-home services. Future studies could assess patients’ perceptions both prior to and after undergoing therapy at a distance as well as assess whether there is indeed a benefit to combining in-person and distance services. Combining in-person and distance services could be done by having one scheduled visit with the therapist or other health professional at the clinical site or patient’s home, such as midway through therapy, although the impact of this remains to be shown.

In general, patients consistently report more positive views of telehealth, including home telecare, than do service providers [22]. Although most studies that examine patient satisfaction with telehealth, including telerehabilitation, report high satisfaction rates, the main area of dissatisfaction reported is generally with technical difficulties [23]. In this study, patients reported that if technical difficulties arose, namely difficulties with establishing a connection between the home and clinical site, this did not impact on their perception of the services received. Indeed, the communication was easily reestablished or the appointment rescheduled. For these participants, this was not problematic as they had limited mobility and were generally available at home. In the first few weeks following a TKA, patients have limited mobility and are greatly limiting their activities, mostly because of the pain [37,38]. This may vary for medical conditions where limited mobility is not a primary concern and/or where the goal is to increase patients’ social participation including returning to work as soon as possible.

### Study Limitations and Future Directions

This study provides a first portrait of telerehabilitation as experienced and perceived by patients who have undergone a TKA. In order to obtain an in-depth understanding of the phenomenon and ensure that different perspectives would be included in the study, the sample included patients with different characteristics that could impact their experiences and perceptions of telerehabilitation (e.g., gender, age, work status, type of housing, living alone or not, internet access that was previously installed or not, functional status prior to surgery). All the patients however received the same telerehabilitation services for the same medical condition, had previously had in-person physiotherapy services, and were recruited from the telerehabilitation arm of an RCT. A sample size of five patients was used since data saturation was achieved regarding their experience with telerehabilitation, with no new themes identified after the third interview; the latter two interviews provided additional examples for themes which had previously been identified. This may in part be due to the similar intervention received by the participants given the nature of their condition and the rigour of the RCT. While results from a case study are not generalizable to all contexts, the extent to which findings from this study are transferable to other settings is increased through a detailed description of the case and context, allowing readers to extract the pertinent information to their setting [39]. This study provided insight into patients’ perceptions based on their experiences. Future studies comparing perceptions of patients receiving different modes of therapy, such as from a distance and in-person would help better understand the role that prior experience plays in the way patients perceive the services they receive. In addition, future studies should explore the relationship between clinical outcome and patient perceptions.

## 5. Conclusions

While it is essential to ensure the efficacy of telerehabilitation interventions, such as through clinical trials, a better understanding of patients’ perceptions with its use is crucial as they are the end-users. This study showed that participants were satisfied with most of the aspects of their experience, including the access to services, the relationship with therapist, the exercises program, the technology and the support provide by the technical team. Given that there are few successfully implemented home telerehabilitation programs, an analysis of patients’ perceptions, as reported here, can be included as part of larger scale studies. Such analyses would provide essential information to support the implementation and sustainability of in-home telerehabilitation programs.
